# Takotsubo syndrome treated with VA-ECMO in plastic surgery: echocardiography first—case series

**DOI:** 10.1093/ehjcr/ytag462

**Published:** 2026-06-22

**Authors:** Anna Greti Everding, Adriana González-Martínez, Cinthya Alejandra Rodríguez-Godínez, Luisa Fernanda Aguilera, Jorge Javier Miguel González, Alex D Pacheco-Bouthillier, Benigno Ferreira-Piña, Oscar Lomelí-Sánchez

**Affiliations:** Cardiovascular Intensive Care Unit and Echocardiography Department, Minimally Invasive Cardiovascular Institute, Blvrd Puerta de Hierro 5090-1er. Piso, Centro Médico Puerta de Hierro, 45116 Zapopan, Jalisco, Mexico; Cardiovascular Intensive Care Unit and Echocardiography Department, Minimally Invasive Cardiovascular Institute, Blvrd Puerta de Hierro 5090-1er. Piso, Centro Médico Puerta de Hierro, 45116 Zapopan, Jalisco, Mexico; Heart Failure Department, Salvando Latidos Foundation, José María Eredia st 2703, 44657 Guadalajara, Jalisco, Mexico; Cardiovascular Intensive Care Unit and Echocardiography Department, Minimally Invasive Cardiovascular Institute, Blvrd Puerta de Hierro 5090-1er. Piso, Centro Médico Puerta de Hierro, 45116 Zapopan, Jalisco, Mexico; Cardiovascular Intensive Care Unit and Echocardiography Department, Minimally Invasive Cardiovascular Institute, Blvrd Puerta de Hierro 5090-1er. Piso, Centro Médico Puerta de Hierro, 45116 Zapopan, Jalisco, Mexico; Heart Failure Department, Salvando Latidos Foundation, José María Eredia st 2703, 44657 Guadalajara, Jalisco, Mexico; Cardiovascular Intensive Care Unit and Echocardiography Department, Minimally Invasive Cardiovascular Institute, Blvrd Puerta de Hierro 5090-1er. Piso, Centro Médico Puerta de Hierro, 45116 Zapopan, Jalisco, Mexico; Cardiovascular Intensive Care Unit and Echocardiography Department, Minimally Invasive Cardiovascular Institute, Blvrd Puerta de Hierro 5090-1er. Piso, Centro Médico Puerta de Hierro, 45116 Zapopan, Jalisco, Mexico; Cardiovascular Intensive Care Unit and Echocardiography Department, Minimally Invasive Cardiovascular Institute, Blvrd Puerta de Hierro 5090-1er. Piso, Centro Médico Puerta de Hierro, 45116 Zapopan, Jalisco, Mexico; Cardiovascular Intensive Care Unit and Echocardiography Department, Minimally Invasive Cardiovascular Institute, Blvrd Puerta de Hierro 5090-1er. Piso, Centro Médico Puerta de Hierro, 45116 Zapopan, Jalisco, Mexico

**Keywords:** Takotsubo cardiomyopathy, Plastic surgery, Extracorporeal membrane oxygenation, Plastic surgery procedures, Case series

## Abstract

**Background:**

Takotsubo syndrome is a transient cardiac disease induced by emotional or physical stress secondary to sympathetic hyperstimulation resulting in regional wall motion abnormalities. Secondary subtypes appear to affect younger patients and reveal in more severe presentations where difficulties in diagnosis and management may arise. Real-world scenarios may significate a literature tool for referral and consideration in certain contexts.

**Case Summary:**

Four previously healthy young women (28, 27, 31, and 34 years old) underwent different plastic surgery procedures, presenting haemodynamic instability in the operating room. Transoesophageal echocardiography showed left ventricular ballooning and decreased systolic function, resembling Takotsubo morphology. All four patients developed refractory cardiogenic shock, and mechanical circulatory support was initiated with later complete left ventricle global mobility normalization and 100% survival.

**Discussion:**

Surgery has been associated as a trigger factor for this syndrome. However, the number of cases related to aesthetic procedures is probably underestimated. Beyond this under-recognition, factors such as the administration of exogenous catecholamines, simultaneous multiple procedures, and prolonged surgical times in patients with an underlying vulnerability may account for the more severe clinical manifestations observed in this setting. Therefore, risk factors screening, prompt identification, and early treatment including mechanical circulatory support, could decrease the mortality rate in these patients. In addition to current algorithms for Takotsubo syndrome, we propose transoesophageal or transthoracic echocardiography as the primary diagnostic tool. Its early use allows prompt recognition and timely intervention, which are key factors for survival in cardiogenic shock.

Learning points

- Plastic surgery procedures may trigger Takotsubo syndrome in susceptible individuals.
- Transthoracic and transoesophageal echocardiography should be considered as a useful first-line diagnostic tool in secondary Takotsubo syndrome in specific scenarios.
- Early mechanical circulatory support may contribute to a reduction in mortality.
- Surgery or stressful events can lead to Takotsubo syndrome.

## Introduction

Takotsubo syndrome (TTS) is an uncommon and often under-recognized acute cardiac condition characterized by regional wall motion abnormalities (RWMA), most frequently involving left ventricular (LV) apical ballooning. Its clinical presentation may mimic acute coronary syndrome or cardiogenic shock (CS), in the absence of obstructive coronary artery disease (CAD).^1,2^ TTS is typically associated with identifiable stressors (‘triggers’), either emotional or physical, which induce sympathetic hyperactivation and catecholamine release, leading to transient myocardial dysfunction and ventricular dilatation (*Summary figure*), with the characteristic morphology resembling the Japanese octopus trap ‘Takotsubo’.^1,3^ The secondary subtype occurs in hospitalized patients following physical triggers.^4^ The estimated incidence is 2–9 per 100 000 persons annually, with up to 1 in 6700 cases occurring in the perioperative setting.^5^ TTS may also develop in apparently healthy young individuals undergoing non-cardiac elective procedures, including aesthetic procedures (AeP).^4,6^ Although generally self-limiting, up to 10% of patients may deteriorate to CS.^7^

Given the low pre-test probability of CAD in young women undergoing elective AeP, this study aimed to describe severe cases of TTS and to highlight the role of an echocardiography-first diagnostic approach in the early identification and management of CS in this setting.

## Summary figure

**Figure:**
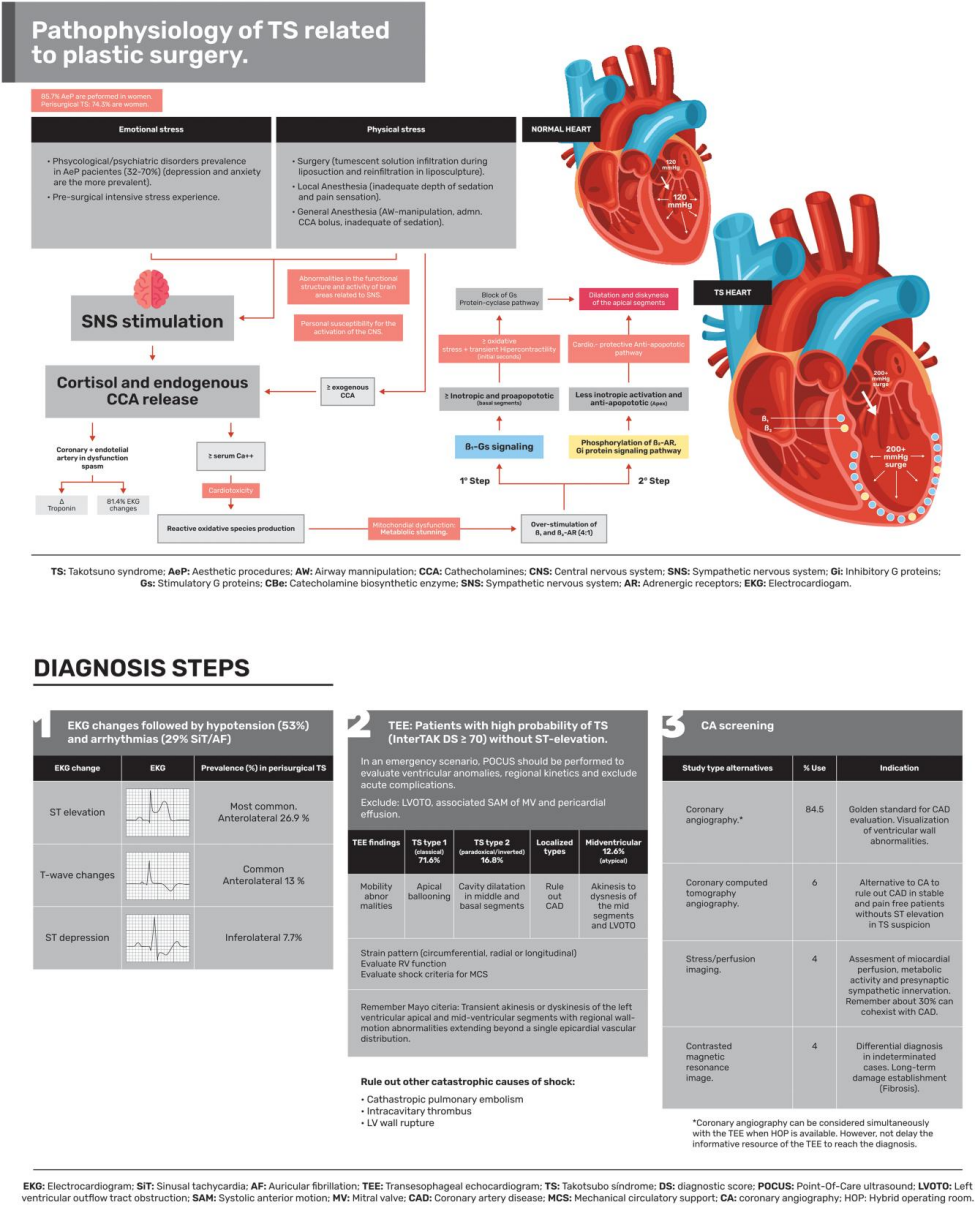
Pathophysiological mechanism of secondary Takotsubo syndrome triggered by aesthetic procedures.

## Case summaries

### Patient 1

A 28-year-old woman (BMI 28), with a history of substance abuse, smoking, and recent use of sibutramine and phentermine (3 weeks prior), underwent elective liposculpture and mammoplasty.

Thoracic paravertebral block with sedation was used. After 90 min, the procedure was discontinued due to hypotension refractory to standard management. ST-segment elevation in leads V2 and V5 was observed, with troponin 0.49 ng/ml and CK-MB 64 ng/ml. Coronary angiography (CAG) showed no significant lesions.

**Figure 1:**
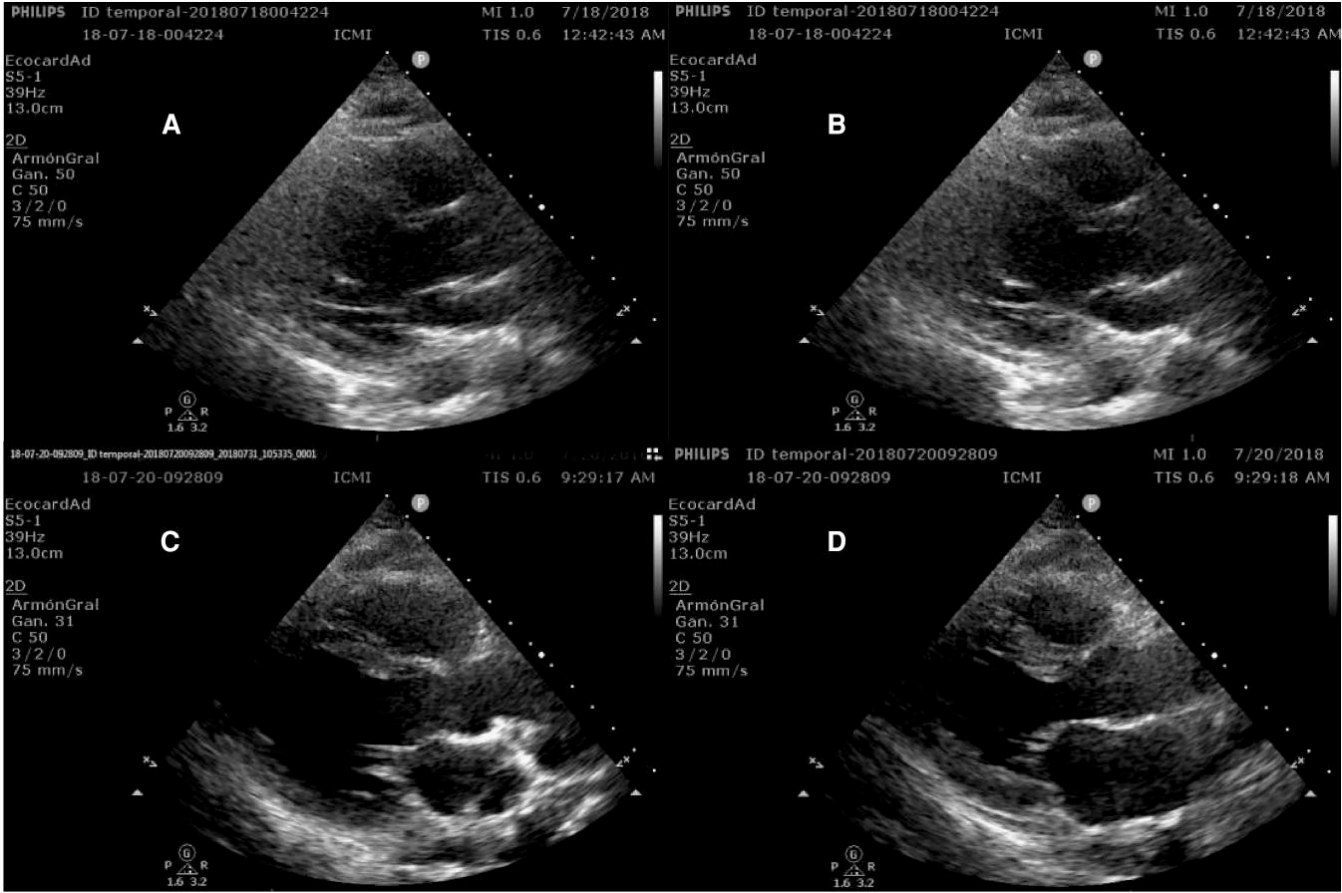
Graphical representation of TTS evolution (Patient 1). Transthoracic echocardiogram with a parasternal view in the acute phase demonstrating severe left ventricular dysfunction (LVFS 10%), ventricular diastole (*A*) and systole (*B*), showing akinesia of the mid and apical segments of the septum and lateral wall. In the recovery phase, improvement of left ventricular function (LVFS 45%) is demonstrated within the following 72 h, diastole (*C*) and systole (*D*).

Given haemodynamic instability and absence of CAD, transthoracic echocardiography (TTE) was prioritized, revealing a left ventricular ejection fraction (LVEF) 10%, LV severe mid-apical hypokinesia (apical ballooning), consistent with type 1 Takotsubo morphology (*Figure 1*, *Table 1*).

**Table 1:** Revised Mayo clinic criteria for TS diagnosis 2008 and echocardiographic data

Mayo criteria	Characteristics presented by the patients			
Cases	1	2	3	4
1) Abnormalities in the movement of the LV wall and/or triggering factor:	Plastic surgery as physical triggering stressor			
LVEF (%) pre ECMO	10	20	30	15
LV linear dimensions:				
LVIDd (mm)	50	48	55	43
LVIDs (mm)	33	30	43	25
IVSd (mm)	8	7	5	7
PWTd (mm)	7	6	5	6
Other parameters of systolic function:				
MAPSE (mm)	5.7	7	4.8	6.3
Mitral annular systolic velocity (cm/s)	8.3	7.3	5.0	7
VTI of LVOT (cm)	6.3	8.0	8.9	7.1
CI (L/min/m^2^)	1.83	2.0	2.22	1.9
2) Absence of obstructive coronary artery disease or acute plaque rupture	Coronary artery angiography without significant lesions	NI	NI	Coronary artery angiography without significant lesions
3) New electrocardiographic abnormalities or modest troponin elevation	Severe ventricular tachycardia and bigeminy.	Severe bradycardia	ST segment depression	AF and VT
	ST segment elevation in V2 and V5			
Troponin I (0.01–0.3 ng/ml)	0.49	0.54	4.12	NI
CPK	NI	317	383	
CK-MB U/L (0–18 U/L)	64	253	39	
4) Pheochromocytoma and myocarditis	Absence	Absence	Absence	Absence

CK-MB, creatine kinase-MB; CI, cardiac index; CPK, creatine phosphokinase; LV, left ventricle; LVEF, left ventricular ejection fraction; LVIDd, left ventricular internal diameter end diastoler; LVIDs, left ventricular internal diameter end systole; LVOT, left ventricular outflow tract; IVSd, inter-ventricular septal thickness in diastole; MAPSE, mitral annular plane systolic excursion; PWTd, posterior wall thickness in diastole; TS, Takotsubo syndrome; VTI, velocity-time integral; VT, ventricular tachycardia.

**Figure 2:**
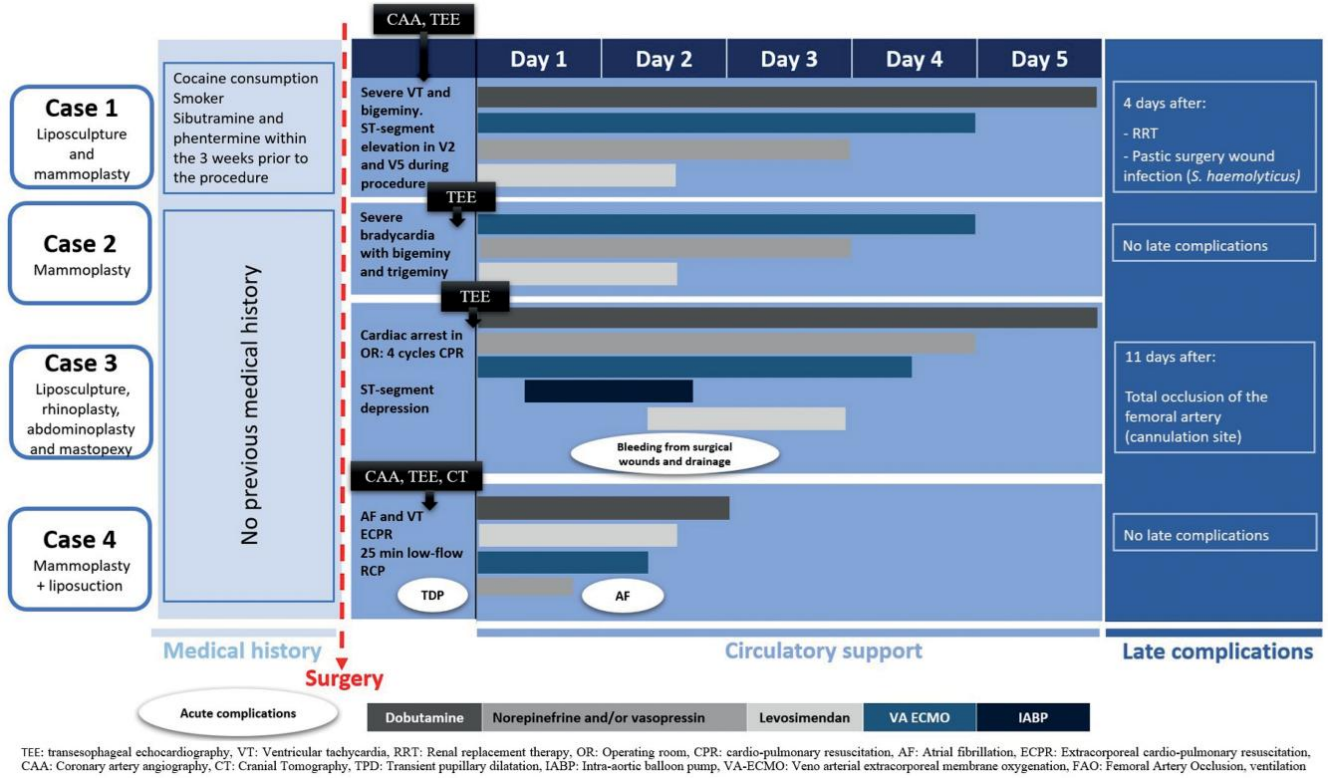
Timeline representation. Including (from left to right): case number and surgery performed, medical history, surgical duration, TEE and/or TTE findings, circulatory support over 5 days, and late complications.

Despite fluid resuscitation, the patient required inotropes and vasopressors, with persistent hypoperfusion. Veno-arterial extracorporeal membrane oxygenation (VA-ECMO) was initiated according to ELSO guidelines and maintained for 4 days without acute complications. The patient regained consciousness within 24 h, required 6 days in the intensive care unit (ICU), and had a total hospital stay of 22 days, prolonged due to *Staphylococcus haemolyticus* wound infection and need for renal replacement therapy (*Figure 2*).

Follow-up TTE at 5 days showed recovery of LVEF to >50% with normal systolic function. The patient was discharged on enalapril and spironolactone. No recurrence was observed over 2 years.

### Patient 2

A 27-year-old woman [body mass index (BMI) 22], with no relevant medical history or identifiable cardiovascular risk factors, was referred to our centre due to haemodynamic deterioration following a mammoplasty procedure. Intraoperatively, she developed severe bradycardia (40 bpm), for which three doses of atropine (1 mg IV) and adrenaline (1 mg IV) were administered. Clinical signs of pulmonary oedema were noted. Given the use of thoracic paravertebral block and sedation, mechanical ventilation was initiated.

Upon admission and given the high clinical suspicion of non-ischaemic CS, coronary angiography was not performed. TTE was therefore selected as the primary diagnostic modality, demonstrating a LVEF of 20% with apical ballooning (*Table 1*). Laboratory findings included troponin of 0.54 ng/ml, creatine phosphokinase (CPK) of 317 U/L, and CK-MB of 253 ng/ml.

Despite optimization of medical therapy, the patient remained in severe CS. Consequently, peripheral VA-ECMO was initiated (treatment described in *Figure 2*).

No early or late complications were observed. The patient required VA-ECMO support for 4 days, with an ICU stay of 7 days and a total hospital stay of 9 days (*Table 2*). Recovery was confirmed at discharge by TTE, demonstrating LVEF >50% with normal global systolic function, and a functional status of NYHA class I (*Figure 1*).

**Table 2:** Patients clinical characteristics and TS Mayo-criteria

Case number	Case 1	Case 2	Case 3	Case 4
Gender	Female	Female	Female	Female
Age	28	27	31	34
Medical history	Cocaine consumption	No	No	No
	Smoker			
	Sibutramine and phentermine within the 3 weeks prior to the procedure			
Intervention	Liposculpture and mammoplasty	Mammoplasty	Liposculpture, rhinoplasty, abdominoplasty and mastopexy	Mammoplasty + liposuction
BMI	28	22	23	23.6
PreECMO cardiac support	Dobutamine	Dobutamine	Dobutamine	Dobutamine
	Levosimendan	Levosimendan		Levosimendan
	Vasopressin	Vasopressin	Vasopressin	Vasopressin
	Norepinephrine	Norepinephrine	Norepinephrine	Norepinephrine
			IABP	
Support	VA-ECMO	VA-ECMO	VA-ECMO	ECPR + VA-ECMO
Days with IMV	1	1	2	0.5
Complications	RRT	None	FAO	AF
	Wound infection of the cosmetic surgery access (*S. haemolyticus*)		Reintervention	Transitory pupillary dilatation
ECMO support days	4	4	4	1
ICU stay	6	6	8	2
Hospital stay	16	8	14	4
Survived	YES	YES	YES	YES
Out hospital treatment	Enalapril and spironolactone	Bad adherence	NI	Dapagliflozin, spironolactone and bisoprolol

AF, atrial fibrillation; BMI, body mass index; ECPR, extracorporeal cardio-pulmonary resuscitation; FAO, femoral artery occlusion, ventilation; IABP, intra-aortic balloon pump; ICU, intensive care unit; IMV, invasive mechanical ventilation; NI, no information; RRT, renal replacement therapy; TS, Takotsubo syndrome; VA-ECMO, veno arterial extracorporeal membrane oxygenation.

### Patient 3

A 31-year-old woman (BMI 23), with no relevant past medical history, developed bradyarrhythmia and ST-segment depression during combined plastic surgical procedures (liposculpture, rhinoplasty, abdominoplasty, and mastopexy). The patient rapidly deteriorated, progressing to cardiac arrest. She subsequently underwent four cycles of advanced cardiopulmonary resuscitation, achieving return of spontaneous circulation.

Urgent TTE demonstrated a LVEF of 30%, with ventricular dilatation and severe basal-to-mid hypokinesia (*Table 1*). Laboratory findings showed mild elevation of cardiac biomarkers, including troponin of 4.12 ng/ml, CPK of 383 U/L, and CK-MB of 39 ng/ml, consistent with a diagnosis of TTS. The patient remained haemodynamically unstable over the subsequent 12 h.

Initial management with vasopressors (*Table 1*) failed to achieve adequate perfusion, and an intra-aortic balloon pump was inserted. However, CS persisted. Arterial blood gas analysis revealed pH 7.32, pO_2_ 165 mmHg, pCO_2_ 34 mmHg, HCO_3_^−^ 16.5 mmol/L, and lactate 6.9 mmol/L, accompanied by tachycardia (135 bpm), hypotension (78/56 mmHg), and oliguria. Consequently, VA-ECMO was initiated. Following haemodynamic stabilization, a levosimendan protocol commenced and completed.

She developed significant bleeding from surgical wounds and liposuction drain sites, requiring politransfusion. Mechanical circulatory support (MCS) was maintained for 4 days (*Table 2*).

Eleven days after decannulation, the patient developed severe pain in the right lower limb. Computed tomography angiography revealed complete occlusion of the right femoral artery. Endovascular angioplasty with endoprosthesis implantation and local thrombolysis was performed, achieving partial recanalization (approximately 40% of the vessel diameter) (*Figure 2*).

Following hospital discharge, the patient underwent 6 months of outpatient physical rehabilitation. Follow-up assessment demonstrated partial recovery, with LVEF of 40% and LV global longitudinal strain of −12.63%, with preserved global wall motion.

### Patient 4

A 34-year-old woman (BMI 23.6), with no prior medical history, underwent liposuction and mammoplasty under subarachnoid blockade (ropivacaine 22.5 mg and hyperbaric bupivacaine 5 mg) combined with thoracic epidural anaesthesia (T4–T5).

During mammoplasty, the patient developed bradyarrhythmia, atrial fibrillation, and ventricular tachycardia, leading to procedure discontinuation. Haemodynamic support with norepinephrine, vasopressin, and dobutamine was initiated, and she was transferred to the hybrid operating room, where she progressed to cardiac arrest. After 25 min of low-flow cardiopulmonary resuscitation, extracorporeal cardiopulmonary resuscitation was initiated. Coronary and pulmonary angiography excluded obstructive coronary artery disease and pulmonary embolism. TTE demonstrated LVEF 15% with severe mid-apical LV dilatation and hypokinesia.

The patient received VA-ECMO for 24 h and amiodarone for atrial fibrillation. Transient pupillary dilatation was observed; cranial computed tomography showed no haemorrhage. Sedation was minimized, and the patient regained consciousness within 5 h without neurological deficits.

Following decannulation, LVEF improved to 50%. She continued a 1-year follow-up in a heart failure clinic with guideline-directed therapy (dapagliflozin, spironolactone, bisoprolol), with NT-proBNP levels of 132 and 60 pg/ml at 2 and 4 months, respectively.

## Discussion

In secondary TTS, triggers induce abrupt sympathetic activation with catecholamine excess, leading to myocardial dysfunction via β_2_-adrenergic receptor signalling and a switch in G-protein coupling from Gs to Gi, resulting in a negative inotropic effect and left ventricular (LV) dysfunction (*Summary figure*).^4,8^ Although TTS predominantly affects postmenopausal women, up to 20% of cases occur in younger patients, typically associated with physical triggers and more severe presentations, consistent with our cohort.^9^

Surgery has been recognized as a significant physical stressor and is strongly associated with the onset of TTS.^8,10^ However, only a limited number of cases have been reported in association with AeP (*Table 3*). In this context, the use of exogenous catecholamines, particularly epinephrine, appears to represent a potential risk factor.^6^ Current liposuction guidelines from the American Society for Dermatologic Surgery recommend a maximum safe dose of 55 mg/kg for tumescent solution.^11^ Higher concentrations of epinephrine (>1 g) have been associated with haemodynamic instability and more severe complications.^12,13^

**Table 3:** List of case reports of TS as complication of plastic surgery procedures

References	Aesthetic procedure	Article type	Age	Gender	Type of anaesthesia	LVEF (%)	TS morphology	Circulatory support	CPR	Survived
Jacob Abraham *et al*. ^6^	Liposuction	Case series	24	Female	1 mg IV epinephrine	15	Type 1	Dopamine	No	Yes
	Face lift		48	Female		35	Type 2	None	No	Yes
Michael Glamore 2012	Rhinoplasty	Case series	18	Female	General and local anaesthesia (2 ml of 2% xylocaine with 1:100 000 epinephrine into the subcutaneous plane above the nasal bones and radix)	15-20	Type 1	NI	No	Yes
			16	Female		NI	NI	None	No	Yes
Sebastiaan Maes *et al*. 2019	Liposuction and fat grafting after autologous breast reconstruction	Case report	50	Female	1 L 0.9% saline solution, 50 ml lidocaine 1% (or 10 mg/ml), 1 ml 1:1000 epinephrine, and 10 ml 8.4% sodium bicarbonate	NI	Type 1	1 mg epinephrine	5 min	Yes
R. Cacdac 2021	Breast implant replacement and Abdominoplasty	Case report	46	Female	Infiltration of 3 bags of 35 mg epinephrine in 1 L of fluid as a tumescent during the abdominoplasty	30–35	NI	Impella CP	No	Yes
								Dobutamine Norepinephrine		
Jose Luis Lopez Valdivia 2022	Liposuction	Case report	24	Female	NI	30	Type 1	Vassopresors levosimendan	3 min	Yes
					Infiltration saline solution 0.9% with epinephrine 1:1,000 000					
Seretis 2022	Two-stage implant-based breast reconstruction with implant and augmentation mastopexy	Case report	42	Female	General anaesthesia	20	Type 2	Epinephrine, levosimendan norepinephrine	No	Yes
					Fentanyl, propofol, and rocuronium for induction and maintainer with sevoflurane and remifentanyl					

CPR, cardiopulmonary resuscitation; IMV, invasive mechanical ventilation; LVEF, left ventricular ejection fraction; NI, no information; TS, Takotsubo syndrome.

Although iatrogenic TTS has been described following high doses of catecholamines, cases have also been reported after administration of standard doses, suggesting an element of individual susceptibility.^6^ Furthermore, Y-Hassan *et al*.^12^ proposed that epinephrine may act as a physical trigger, inducing TTS through sympathetic hyperactivation.

Psychiatric disorders have also been identified as a risk factor for TTS.^8^ While none of our patients reported a prior psychiatric diagnosis, formal preoperative psychiatric assessment was not performed. Notably, a systematic review by Jang *et al*.^14^ estimated that up to 40% of patients undergoing elective AeP may have underlying psychiatric conditions, including anxiety and depressive disorders. Regardless of psychiatric history, it is essential that surgeons and the perioperative team consider TTS in patients undergoing AeP who develop early haemodynamic instability, arrhythmias, low cardiac output syndrome, or CS.

We hypothesize that TTS in the context of AeP may be under-recognized. According to the International Society of Aesthetic Plastic Surgery, the number of aesthetic procedures has increased by approximately 41.3% over the past 4 years, with nearly 30% performed in office-based settings.^15^ Supporting these data, Seong Soon Kwon *et al*.^16^ identified more than 200 office-based cosmetic surgery clinics in proximity to their institution and reported 31 cases of cardiac arrest over a 7-year period in patients undergoing AeP. Notably, no systematic screening for TTS was performed in these cases.

TTS remains a diagnosis primarily based on exclusion and clinical features, and failure to fulfil all criteria may result in diagnostic uncertainty.^4,17^ In this setting, an echocardiography-first approach is particularly valuable in young patients undergoing AeP who develop CS, given their low pre-test probability of CAD. Bedside echocardiography allows rapid assessment of ventricular function, identification of RWMA patterns, and exclusion of alternative diagnoses, facilitating early decision-making and timely escalation to MCS. Although guidelines recommend coronary angiography in ST-elevation scenarios, early echocardiographic evaluation in selected patients may expedite diagnosis without delaying life-saving interventions.^3,4,8^

Current ESC consensus considers CAG with left ventriculography the gold standard, particularly in patients with ST-segment elevation or a low InterTAK score.^3,8^ It does not adequately address perioperative or CS settings, where sedation, limited clinical data, and time constraints challenge decision-making.^18^ In our series, patients fulfilled most—but not all—criteria (*Table 1*), all presenting with electrocardiographic abnormalities and severe ventricular dilatation: three with classical apical ballooning and one with reverse basal morphology.

Notably, incomplete recovery of LV function in Patient 3 is atypical for TTS and may reflect the heterogeneous clinical spectrum of the condition. Although TTS has traditionally been considered a transient and fully reversible cardiomyopathy, increasing evidence suggests that some patients may experience delayed recovery, residual ventricular dysfunction, or persistent symptoms during follow-up.

In this context, the absence of cardiac magnetic resonance imaging limits diagnostic certainty, particularly regarding tissue characterization and exclusion of alternative diagnoses such as myocarditis or underlying cardiomyopathy. Echocardiography remains essential in the acute perioperative setting because of its immediate availability and ability to identify characteristic wall-motion abnormalities; however, atypical cases or incomplete recovery patterns may benefit from complementary cardiac MRI assessment for myocardial oedema, inflammation, fibrosis, and right ventricular involvement. Therefore, patients with persistent ventricular dysfunction may require close long-term follow-up and repeat imaging assessment to guide prognosis and medical management.

Secondary TTS is associated with higher complication rates, morbidity, and mortality, particularly in the presence of CS. Identified risk factors include younger age, physical stressors, reduced LVEF, diabetes mellitus, atypical TTS morphology, and atrial fibrillation.^7^ Between 6% and 20% of patients may develop CS through different mechanisms.^7,19^ In our series, extensive RWMA leading to pump failure and refractory shock was a consistent finding. These observations support early consideration of MCS, particularly VA-ECMO, as a bridge to recovery.

Importantly, catecholamine-based inotropic support may exacerbate the underlying pathophysiology and is therefore discouraged.^8^ In our patients, vasopressors and inotropes were initially required; however, prompt escalation to MCS, ensured adequate end-organ perfusion and facilitated myocardial recovery. Levosimendan was administered early during MCS (0.2 µg/kg/min over 24 h) as a non-catecholaminergic inotrope. While it may improve haemodynamic parameters, its vasodilatory effects necessitate cautious use, and current evidence does not demonstrate a consistent mortality benefit in CS.^20^

Taken together, our findings support the use of TTE as first-line diagnostic tools in selected high-risk scenario, namely young patients with low cardiovascular risk, suspected secondary TTS, perioperative onset, and severe haemodynamic compromise (*Summary figure*). In such settings, prioritizing echocardiography may enable rapid diagnosis and early initiation of MCS, without unnecessary delays associated with routine CAG, which may not always be immediately feasible.

This case series includes only patients with severe presentations requiring MCS. Less severe forms of TTS may have occurred but were not systematically captured, introducing potential selection bias.

## Conclusion

Takotsubo syndrome in the setting of AeP may be under-recognized, particularly in young patients presenting with CS. In this context, our findings support an echocardiography-first approach in carefully selected patients, allowing rapid diagnosis and facilitating early, goal-directed management. Importantly, timely identification may enable prompt initiation of MCS, including VA-ECMO, as a bridge to recovery in refractory cases. Further studies are warranted to establish tailored diagnostic algorithms and management strategies for this high-risk perioperative population.

## Lead author biography


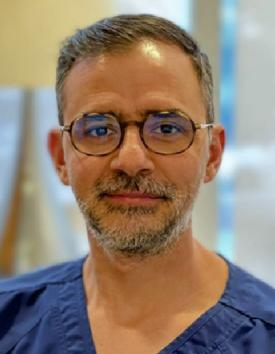
Cardiologist with 8 years of experience, currently practicing in the private practice (ICMI) and at Hospital Civil de Guadalajara (Fray Antonio Alcalde). Specializes in cardiac critical care, managing patients with ECMO support and post-operative care following conventional cardiac surgery, MICs, and aortic surgeries, including hybrid interventions like MIDCAB and TAVI. Skilled in ICU procedures, particularly proficient in echocardiography, becoming a key element in the heart team for echocardiographic assessment, including stress, interventional, and transoesophageal echocardiography, exclusively in adult patients. Also serves as a professor at the Universidad de Guadalajara and Tecnologico de Monterrey in cardiology and critical care.

## Data Availability

The data that support the findings of this case series are available from the corresponding author upon reasonable request.
